# The kSORT Assay to Detect Renal Transplant Patients at High Risk for Acute Rejection: Results of the Multicenter AART Study

**DOI:** 10.1371/journal.pmed.1001759

**Published:** 2014-11-11

**Authors:** Silke Roedder, Tara Sigdel, Nathan Salomonis, Sue Hsieh, Hong Dai, Oriol Bestard, Diana Metes, Andrea Zeevi, Albin Gritsch, Jennifer Cheeseman, Camila Macedo, Ram Peddy, Mara Medeiros, Flavio Vincenti, Nancy Asher, Oscar Salvatierra, Ron Shapiro, Allan Kirk, Elaine Reed, Minnie M. Sarwal

**Affiliations:** 1 Department of Surgery, University of California San Francisco, San Francisco, California, United States of America; 2 Biomedical Informatics, Cincinnati Children's Hospital Medical Center, Cincinnati, Ohio, United States of America; 3 California Pacific Medical Center, San Francisco, California, United States of America; 4 Renal Transplant Unit, Bellvitge University Hospital, Barcelona, Spain; 5 Thomas E. Starzl Transplantation Institute, University of Pittsburgh, Pittsburgh, Pennsylvania, United States of America; 6 Immunogenetics Center, University of California Los Angeles, Los Angeles, California, United States of America; 7 Department of Surgery, Emory University, Atlanta, Georgia, United States of America; 8 Laboratorio de Investigacion en Nefrologia, Hospital Infantil de México Federico Gómez, Mexico City, Mexico; 9 Stanford University, Stanford, California, United States of America; Istituto Mario Negri, Italy

## Abstract

**Background:**

Development of noninvasive molecular assays to improve disease diagnosis and patient monitoring is a critical need. In renal transplantation, acute rejection (AR) increases the risk for chronic graft injury and failure. Noninvasive diagnostic assays to improve current late and nonspecific diagnosis of rejection are needed. We sought to develop a test using a simple blood gene expression assay to detect patients at high risk for AR.

**Methods and Findings:**

We developed a novel correlation-based algorithm by step-wise analysis of gene expression data in 558 blood samples from 436 renal transplant patients collected across eight transplant centers in the US, Mexico, and Spain between 5 February 2005 and 15 December 2012 in the Assessment of Acute Rejection in Renal Transplantation (AART) study. Gene expression was assessed by quantitative real-time PCR (QPCR) in one center. A 17-gene set—the Kidney Solid Organ Response Test (kSORT)—was selected in 143 samples for AR classification using discriminant analysis (area under the receiver operating characteristic curve [AUC] = 0.94; 95% CI 0.91–0.98), validated in 124 independent samples (AUC = 0.95; 95% CI 0.88–1.0) and evaluated for AR prediction in 191 serial samples, where it predicted AR up to 3 mo prior to detection by the current gold standard (biopsy). A novel reference-based algorithm (using 13 12-gene models) was developed in 100 independent samples to provide a numerical AR risk score, to classify patients as high risk versus low risk for AR. kSORT was able to detect AR in blood independent of age, time post-transplantation, and sample source without additional data normalization; AUC = 0.93 (95% CI 0.86–0.99). Further validation of kSORT is planned in prospective clinical observational and interventional trials.

**Conclusions:**

The kSORT blood QPCR assay is a noninvasive tool to detect high risk of AR of renal transplants.

*Please see later in the article for the Editors' Summary*

## Abstract 2

Minnie Sarwal and colleagues developed a gene expression assay using peripheral blood samples to detect patients with renal transplant at high risk for acute rejection.

*Please see later in the article for the Editors' Summary*

## Editors' Summary

**Background:**

Throughout life, the kidneys filter waste products (from the normal breakdown of tissues and food) and excess water from the blood to make urine. If the kidneys stop working for any reason, the rate at which the blood is filtered decreases, and dangerous amounts of creatinine and other waste products build up in the blood. The kidneys can fail suddenly (acute kidney failure) because of injury or poisoning, but usually failing kidneys stop working gradually over many years (chronic kidney disease). Chronic kidney disease is very common, especially in people who have high blood pressure or diabetes and in elderly people. In the UK, for example, about 20% of people aged 65–74 years have some degree of chronic kidney disease. People whose kidneys fail completely (end-stage kidney disease) need regular dialysis (hemodialysis, in which blood is filtered by an external machine, or peritoneal dialysis, which uses blood vessels in the abdominal lining to do the work of the kidneys) or a renal transplant (the surgical transfer of a healthy kidney from another person into the patient's body) to keep them alive.

**Why Was This Study Done?:**

Our immune system protects us from pathogens (disease-causing organisms) by recognizing specific molecules (antigens) on the invader's surface as foreign and initiating a sequence of events that kills the invader. Unfortunately, the immune system sometimes recognizes kidney transplants as foreign and triggers transplant rejection. The chances of rejection can be minimized by “matching” the antigens on the donated kidney to those on the tissues of the kidney recipient and by giving the recipient immunosuppressive drugs. However, acute rejection (rejection during the first year after transplantation) affects about 20% of kidney transplants. Acute rejection needs to be detected quickly and treated with a short course of more powerful immunosuppressants because it increases the risk of transplant failure. The current “gold standard” method for detecting acute rejection if the level of creatinine in the patient's blood begins to rise is to surgically remove a small piece (biopsy) of the transplanted kidney for analysis. However, other conditions can change creatinine levels, acute rejection can occur without creatinine levels changing (subclinical acute rejection), and biopsies are invasive. Here, the researchers develop a noninvasive test for acute kidney rejection called the Kidney Solid Organ Response Test (kSORT) based on gene expression levels in the blood.

**What Did the Researchers Do and Find?:**

For the Assessment of Acute Rejection in Renal Transplantation (AART) study, the researchers used an assay called quantitative polymerase chain reaction (QPCR) to measure the expression of 43 genes whose expression levels change during acute kidney rejection in blood samples collected from patients who had had a kidney transplant. Using a training set of 143 samples and statistical analyses, the researchers identified a 17-gene set (kSORT) that discriminated between patients with and without acute rejection detected by kidney biopsy. The 17-gene set correctly identified 39 of the samples taken from 47 patients with acute rejection as being from patients with acute rejection, and 87 of 96 samples from patients without acute rejection as being from patients without acute rejection. The researchers validated the gene set using 124 independent samples. Then, using 191 serial samples, they showed that the gene set was able to predict acute rejection up to three months before detection by biopsy. Finally, the researchers used 100 blood samples to develop an algorithm (a step-wise calculation) to classify patients as being at high or low risk of acute rejection.

**What Do These Findings Mean?:**

These findings describe the early development of a noninvasive tool (kSORT) that might, eventually, help clinicians identify patients at risk of acute rejection after kidney transplantation. kSORT needs to be tested in more patients before being used clinically, however, to validate its predictive ability, particularly given that the current gold standard test against which it was compared (biopsy) is far from perfect. An additional limitation of kSORT is that it did not discriminate between cell-mediated and antibody-mediated immune rejection. These two types of immune rejection are treated in different ways, so clinicians ideally need a test for acute rejection that indicates which form of immune rejection is involved. The authors are conducting a follow-up study to help determine whether kSORT can be used in clinical practice to identify acute rejection and to identify which patients are at greatest risk of transplant rejection and may require biopsy.

**Additional Information:**

Please access these websites via the online version of this summary at http://dx.doi.org/10.1371/journal.pmed.1001759.

- The US National Kidney and Urologic Diseases Information Clearinghouse provides links to information about all aspects of kidney disease; the US National Kidney Disease Education Program provides resources to help improve the understanding, detection, and management of kidney disease (in English and Spanish)
- The UK National Health Service Choices website provides information for patients on chronic kidney disease and about kidney transplants, including some personal stories
- The US National Kidney Foundation, a not-for-profit organization, provides information about chronic kidney disease and about kidney transplantation (in English and Spanish)
- The not-for-profit UK National Kidney Federation provides support and information for patients with kidney disease and for their carers, including information and personal stories about kidney donation and transplantation
- World Kidney Day, a joint initiative between the International Society of Nephrology and the International Federation of Kidney Foundations, aims to raise awareness about kidneys and kidney disease
- MedlinePlus provides links to additional resources about kidney diseases, kidney failure, and kidney transplantation; the MedlinePlus encyclopedia has a page about transplant rejection

## Introduction

Despite the application of high-throughput discovery methods for improved detection of disease-specific biomarkers [1]–[4], there is ambiguity about the path to developing a clinically applicable molecular assay with sufficiently high sensitivity and specificity, such that it can be used in practice for disease diagnosis and patient care management. In kidney transplantation, acute rejection (AR) occurs in about 15%–20% of patients using the current standard of care for immunosuppression and is detected by an invasive biopsy following a drift in the patient's serum creatinine, the current peripheral marker for graft injury. However, a drift in serum creatinine is nonspecific for AR and occurs only after substantive graft damage. In addition, in a number of patients, AR occurs subclinically, without a drift in serum creatinine [5], and thus these AR cases remain largely undetected until extensive graft damage develops [6]. Both AR and subclinical AR represent major risk factors for developing chronic graft injury and graft loss, which not only require cost-intensive patient care (with dialysis costs of up to US$100,000/year per person in the US) but also reduce the quality of life of patients. Some transplant programs perform protocol biopsies as a means of frequent graft monitoring, but thereby increase the risk of unnecessary invasive procedures in patients who do not have AR, and add to the financial burden of insurance payers. Furthermore, biopsy histology is subject to sampling error [7],[8] and is not predictive of AR. The development of a sensitive, specific, and noninvasive test for the risk of AR is therefore a critical and currently unmet need in transplantation. In the present study we developed a simple blood quantitative real-time PCR (QPCR) test called the Kidney Solid Organ Response Test (kSORT) to predict AR, improve risk stratification assessment in transplantation, and provide relevant information for immunosuppression decision-making [9],[10].

## Methods

### Ethics Statement

All patients gave written informed consent to participate in the study, and the study was approved by the individual institutional review boards at Stanford University, California Pacific Medical Center, Emory University, University of Pittsburgh Medical Center, University of California Los Angeles, University of California San Francisco, Bellvitge University Hospital, and the Hospital Infantil de México Federico Gómez. All procedures were conducted according to the principles expressed in the Declaration of Helsinki [11].

### Study Design

**Figure 1:**
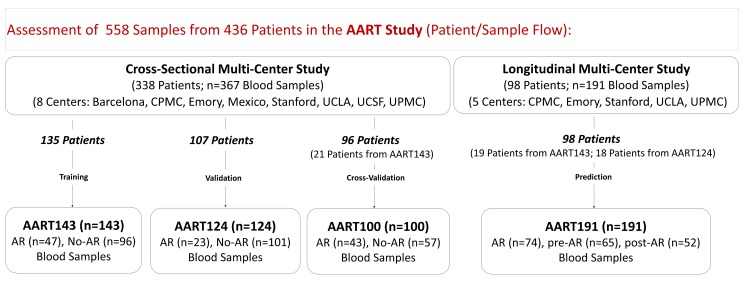
AART study design in 436 unique adult/pediatric renal transplant patients from eight transplant centers. 558 PB samples from 436 adult and pediatric renal transplant patients collected from eight independent transplantation centers in the US, Spain, and Mexico were assessed. Emory, UCLA, UPMC, CPMC, UCSF, and Barcelona contributed adult samples, Mexico and Stanford, pediatric samples. For kSORT QPCR analysis, cross-sectional AART samples were divided into three cohorts: AART143, *n* = 143 adult samples from 135 patients for kSORT gene modeling; AART124, *n* = 124 independent adult (*n* = 59) and pediatric (*n* = 65) samples from 107 adult (*n* = 52) and pediatric (*n* = 55) patients (for independent kSORT validation); AART100, *n* = 100 adult (*n* = 78) and pediatric (*n* = 22) samples from 96 adult (*n* = 75, of which 21 patients were also included in AART143) and pediatric (*n* = 21) patients for final kSORT assay lock and clinical translation. For kSORT QPCR analyses, longitudinal samples were included in AART191: *n* = 191 adult (*n* = 94) and pediatric (*n* = 97) serial samples from 98 adult (*n* = 58) and pediatric (*n* = 40) patients for kSORT prediction. Detailed demographic patient information is shown in Table 1; detailed sample and patient flow is outlined in Table S2.

The Assessment of Acute Rejection in Renal Transplantation (AART) study was designed in a collaborative effort in eight renal transplant centers in the United States, Mexico, and Spain, and utilized 558 peripheral blood (PB) samples from 436 adult and pediatric renal transplant patients for developing a simple blood QPCR test for AR diagnosis and prediction in recipients of diverse ages, on diverse immunosuppression, and subject to transplant-center-specific protocols. Patients <20 y of age were included in the pediatric cohort, and patients ≥20 y were included in the adult cohort. The mean age in the pediatric dataset was 13.20 y±4.76 y, and the mean age in the adult dataset was 47.43 y±14.59 y. Blood samples were collected from transplant recipients cross-sectionally during clinical follow-up visits and were matched with a contemporaneous kidney allograft biopsy between 2005 and 2012. Centers that participated in the AART study were Stanford University, Stanford, California (Stanford; *n* = 162 pediatric samples); Hospital Infantil de México Federico Gómez Laboratorio de Investigacion en Nefrologia, Mexico City, Mexico (Mex; *n* = 23 pediatric samples); Emory University, Atlanta, Georgia (Emory; *n* = 43 adult samples); University of California Los Angeles, Los Angeles, California (UCLA; *n* = 105 adult samples); University of Pittsburgh, Pittsburgh, Pennsylvania (UPMC, *n* = 132 adult samples); California Pacific Medical Center, San Francisco, California (CPMC; *n* = 37 adult samples); University of California San Francisco, San Francisco, California (UCSF; *n* = 40 adult samples); and Bellvitge University Hospital, Barcelona, Spain (Barcelona; *n* = 16 samples). Samples were split into a training set of 143 AR and No-AR adult samples collected in non-clinical-trial settings in four centers (AART143 cohort) for gene selection and model training; a first validation set of 124 independent AR and No-AR adult (≥20 y) and pediatric (<20 y) samples (AART124 cohort) collected by each participating center, for validation of selected genes for AR detection across ages, centers, and settings; and a second prospective validation set of 191 adult and pediatric samples serially collected up to 6 mo prior to and after a rejection biopsy (AART191 cohort) for evaluation of AR prediction. Blood samples from these three cohorts were simultaneously measured on the microfluidic high-throughput BioMark QPCR platform (Fluidigm, South San Francisco, CA) [12] for a total of 43 genes. The final kSORT assay of 17 genes for noninvasive detection of AR was locked in a third validation set of 100 adult and pediatric samples (AART100 cohort) on the Applied Biosystems (ABI; Foster City, CA) QPCR platform with the development of a novel algorithm (kSORT analysis suite [kSAS]). 21 patients in the AART143 cohort contributed samples to the AART100 cohort (Figure 1; Tables 1 and S2). Histology readings of the matched biopsies were not assessed in a centralized manner.

**Table 1:** Demographics of unique patients included in the AART Study.

Parameters	Transplant Center							
	Stanford	Mexico	UCLA	Emory	UPMC	CMPC	UCSF	Barcelona
**Total number of patients**	96	19	53	35	86	33	40	16
**Number with AR biopsy**	46	11	37	13	19	2	7	8
**Mean (SD) donor age (years)**	26.7 (9.5)	37.1 (12.8)	38.4 (13.2)	38.3 (12.2)	50.1 (11.1)	—	33.8 (11.9)	43.4 (12.9)
**Donor gender**								
Percent male	56.36%	57.14%	50.00%	59.09%	44.44%	—	47.62%	—
Number male/total	28/55	8/14	12/24	13/22	32/72	—	10/21	—
**Transplant type**								
Percent deceased	63.6%	35.7%	45.8%	68.8%	9.7%	42.4%	51.9%	92.9%
Number deceased/total	35/55	5/14	11/24	22/32	7/72	14/33	14/27	13/14
**Mean (SD) recipient age (years)**	13.2 (4.8)	15.0 (1.8)	44.3 (12.0)	45.2 (16.1)	49.2 (15.0)	52.8 (18.2)	54.6 (11.9)	46.0 (12.9)
**Recipient gender**								
Percent male	50.9%	85.7%	70.8%	66.7%	59.7%	57.6%	60.0%	50.0%
Number male/total	28/55	12/14	17/24	22/33	43/72	19/33	24/40	8/16
**Mean (SD) HLA mismatch (** ***x*** **/6)**	3.1 (1.5)	1.9 (1.5)	3.9 (1.5)	—	3.5 (1.8)	—	4.2 (1.9)	3.9 (0.8)
**Induction**	Dac	Basi/Dac	Thymo	Thymo	Anti-CD52	Thymo	Thymo	Thymo
**Primary IS**	CNI, MMF, ±CS	CNI, MMF, ±CS	CNI, MMF, CS	CNI, MMF, CS	CNI, MMF	CNI, MMF, CS	CNI, MMF, CS	CNI, MMF, CS
**Blood collection**	PAXgene	PAXgene	PBMC	PBMC	PBL	PAXgene	PBMC	PAXgene
**Centralized SOP**	Yes	No	No	No	No	No	No	No
**Centralized RNA extraction**	Yes	No	No	Yes	No	Yes	Yes	No

Means and standard deviations were calculated for patients with clinical data available.

Basi, basiliximab; CS, corticosteroids; Dac, daclizumab; MMF, mycophenolate mofetil; SD, standard deviation; SOP, standard operating procedure; Thymo, thymoglobulin.

### Patient and Sample Information

All adult patients included in this study were enrolled in the regular transplant program at each center. Pediatric patients were those participating in an investigator-initiated clinical trial [13] who also gave written informed consent to participate in this study. Patients did not undergo additional study-specific procedures, and no adverse events were reported. Each PB sample analyzed in this study was paired with a contemporaneous renal allograft biopsy (within 48 h) from the same patient, in all cases of AR (*n* = 188) and all cases of No-AR (*n* = 370), with the exception of 12 samples collected before or after a rejection and 13 samples from clinically stable patients (stable defined as absence of a drift in serum creatinine >20% of baseline, no donor-specific antibodies [DSA] and no rejection within 3 mo post-transplantation). Blood samples collected at the time of clinically indicated biopsies (drift in serum creatinine >20% of baseline) were included in the analyses if obtained prior to any immunosuppression treatment intensification. Blood samples collected at the point of protocol biopsies were obtained from all pediatric patients at engraftment; at 3, 6, 12 and 24 mo post-transplantation; and additionally at the times of clinically suspected graft dysfunction [2]. In the adult cohort the majority of patients had clinically indicated biopsies only for allograft dysfunction; protocol biopsies in the adult cohort were obtained from patients enrolled in Barcelona (*n* = 16) at 6 mo post-transplantation. Multiple PB–biopsy pairs from the same patient were utilized as long as each biopsy had a conclusive phenotypic diagnosis. Each biopsy was scored by the center pathologist at each enrolling clinical site using the latest Banff classification criteria for renal allograft pathology [14]. The blood–biopsy pairs were categorized as AR (*n* = 188) or No-AR (*n* = 370). AR included both cellular and humoral types and was defined as presence of (1) acute interstitial and tubular Banff scores ≥1 or (2) lesions in the glomeruli and peritubular capillary compartments paired with diffuse positive C4d staining at peritubular capillaries (humoral AR). No-AR was defined as an absence of any histological evidence of AR. In the No-AR group, there were 40 samples that either met the Banff criteria for chronic allograft injury (samples displaying interstitial fibrosis/tubular atrophy scores ≥1; *n* = 3) or exhibited chronic calcineurin inhibitor (CNI) toxicity (*n* = 6), BK polyomavirus (BKV) infection (*n* = 4), or other graft injury such as acute tubular nephritis (*n* = 27). While 19 cases met the criteria of humoral rejection, the remaining AR biopsies were considered cell-mediated rejection or mixed rejection if they had absent C4d staining but detectable DSA. Samples included in the AART study were collected between 5 February 2005 and 15 December 2012.

### Sample Collection and Processing

Blood was collected in 2.5-ml PAXgene Blood RNA Tubes (PreAnalytiX, Qiagen, Valencia, CA), in Ficoll tubes for PB lymphocyte (PBL) isolation, or in heparin-coated tubes for PB mononuclear cell (PBMC) isolation. Total RNA was extracted using the column-based method kits of PreAnalytiX (Qiagen) for PAXgene tubes and RNeasy (Qiagen) for PBLs and PBMCs as per manufacturer's protocol. Total RNA was measured for RNA integrity using the RNA 6000 Nano LabChip Kit on a 2100 Bioanalyzer (both from Agilent Technologies, Santa Clara, CA), with suitable RNA defined by an RNA integrity number exceeding 7 [15],[16].

### Quantitative Polymerase Chain Reaction

#### cDNA synthesis

cDNA synthesis was performed using 250 ng of extracted quality mRNA from the PB samples using the SuperScript II first strand cDNA synthesis kit (Invitrogen, Carlsbad, CA) as per the manufacturer's protocol.

#### Total RNA sample preparation for microfluidic QPCR

Samples were prepared for microfluidic QPCR analysis of 43 genes by taking 1.52 ng of total RNA from cDNA synthesis and processing it through specific target amplification and sample dilution as described by Fluidigm using pooled individual ABI Taqman assays for each gene, excluding ribosomal 18S RNA. Briefly, specific target amplification was performed using 1.52 ng of cDNA with the pooled Taqman assays in multiplex with Taqman PreAmp Master Mix (ABI) to 5 µl of final volume, for 18 cycles in a Vapo-Protect thermal cycler (Eppendorf, Hamburg, Germany), then diluted 1∶5 with sterile water (Gibco, Invitrogen, Carlsbad, CA).

#### Microfluidic QPCR

Microfluidic QPCR was performed on the 96.96 dynamic array (Fluidigm) using 2.25 µl of the diluted cDNA sample from the specific target amplification, along with Taqman assays for each gene, Taqman Universal Master Mix, and Fluidigm loading reagent as outlined in the manufacturer's protocol, by priming and loading the chip via the HX IFC Controller (Fluidigm) and performing the QPCR in the BioMark QPCR platform (Fluidigm), with default parameters for gene expression data collection as advised by Fluidigm.

#### ABI QPCR

Standard protocols were used for QPCR reactions on the ABI 7900 Sequence Detection System or the ViiA7 (ABI) under standard conditions (10 min at 95°C, 40 cycles of 15 s at 95°C, 30 s at 60°C), using the same TaqMan gene expression assays (ABI) utilized in the Fluidigm QPCR.

### Statistical Analysis

#### QPCR data preprocessing and normalization

The relative amount of mRNA expression in each sample was calculated using the comparative threshold cycle (C_T_) method [12]. Expression values for all genes were normalized to ribosomal 18S RNA (*18S*) as *18S* showed the least variability in gene expression across all samples when compared to the expression of three additional housekeeping genes tested (*GAPDH, B2M, ACTB*). Visualization of the raw QPCR data and identification of external confounders were done in Partek Genomics Suite version 6.6 (Partek, St. Louis, MO) by unsupervised principal component analysis, hierarchical clustering, and analysis of covariance, and in R by the Empirical Bayes method, which uses estimations for the least squares parameters (mean and variance) for each gene (ComBat method using the SVA package in R) [17]. RNA source (PBMCs, PBLs, or PAXGene), blood sample collection (non-standardized non-clinical-trial versus standardized clinical trial collection protocols), and patient age group (pediatric or adult) were significantly associated with differential gene expression between AR and No-AR groups by analysis of variance (ANOVA) (*p*<0.05) and were thus included as random categorical factors in the batch removal feature (mixed effect model ANOVA) provided in Partek Genomics Suite for additional data normalization applied to the Fluidigm QPCR data, where factors were chosen at random to correct for an overall variability associated with these factors [18] (Figure S1).

#### Selection of 43 genes for AR screening

The 43-rejection-gene set represents a combination of significant genes in acute renal allograft rejection identified by whole genome microarray analysis of biopsy and paired blood samples transcriptionally profiled from patients with and without biopsy-confirmed AR. The discovery process for these genes has been previously published by us [2],[19]. Ten of the 43 rejection genes (*CFLAR, DUSP1, IFNGR1, ITGAX, MAPK9, NAMPT, NKTR, PSEN1, RNF130, RYBP*) were significantly associated with AR in the pediatric dataset analyzed at Stanford and cross-validated in the National Institutes of Health (NIH) SNSO1 randomized multicenter trial [13]. An additional 14 genes (*ABTB1, ANK1, CEACAM4, CHST11, EPOR, IL2RB, MAP2K3, NFE2, PCTP, PSMB9, RUNX3, SLC25A37, STAT3, YPEL3*) were selected from our initial blood microarray discovery performed across three different microarray platforms [2] for having significant differential expression in AR (significance analysis of microarray false discovery rate <5%). As our previous studies in AR have consistently indicated that the AR gene signature in blood is enriched in infiltrated leukocytes, particularly trafficking monocytes, we further included in the 43-rejection-gene set genes with enrichment in trafficking monocytes (*C1orf38, GBP1, GBP2, LYST, RARA, RXRA, TNFRSF1A*); natural killer cells, T lymphocytes, and dendritic cells (*PFN1, ADAM8, RHEB, GZMK*); and endothelial cells (*MMP1*); significant enrichment was determined as >3-fold increase above the mean expression in all cells (using the GSE1133 dataset). Remaining genes were selected based on other studies by our group on renal transplant AR (*IL7, IL7R, FOXP3, GZB*; [1]) and in AR across organs (*CXCL10, ISG20, INPP5D*; [3]). Four different housekeeping genes were selected (*18S, B2M, GAPDH, ACTB*).

#### Selection of 17 genes for kSORT in Fluidigm QPCR data

First, we evaluated the differential expression of ten out of 43 genes, selecting the same ten genes (*CFLAR, DUSP1, IFNGR1, ITGAX, MAPK9, NAMPT, NKTR, PSEN1, RNF130, RYBP*) that have been validated as diagnostic for AR in PB using standardized clinical trial sample collection and processing protocols [2]. The validation of the excellent discriminatory potential of these genes for biopsy-confirmed AR was conducted in a multicenter, US, randomized prospective clinical trial (SNSO1), supported by the NIH National Institute of Allergy and Infectious Diseases, in which a minimum set of different combinations of five of the ten genes were sufficient to detect AR in the graft [2],[5],[13]. These same ten genes were evaluated for their ability to classify AR in a cohort of AR samples collected only from adult renal transplant recipients (AART143), allowing for inclusion of samples not collected under the same strict protocol guidelines as used in the pediatric study [2]. To improve the diagnostic accuracy for AR in the 143 adult samples, various statistical modeling methods were used to include an additional seven genes (*CEACAM4, EPOR, GZMK, RARA, RHEB, RXRA, SLC25A3*7), which improved the accuracy of the gene panel for discriminating AR from No-AR samples, for a final selection of 17 genes. These 17 genes were then validated for their discriminatory performance in an independent cohort of both adult and pediatric biopsy-matched blood samples (AART124) collected by non-standardized non-clinical-trial methods as well as by standardized clinical trial methods. The statistical methods used for selection of the 17 genes included *t*-test statistics with the Bonferroni post-correction or the *q*-value method to control the false discovery rate (for differential gene expression analysis between AR and No-AR); penalized logistic regression with the lasso and elastic net regularization paths (to define genes with the highest predictive power for AR) [20],[21]; regularization paths for generalized linear models via coordinate descent for estimations for penalized logistic regression, available through the R package glmnet [20]; and partial least squares discriminant analysis (plsDA) (for gene selection with discriminant analyses and equal prior probability for classification) [22], available through Partek Genomics Suite version 6.6. For each bootstrap a nested cross-validation loop estimated the best value for lambda according to the deviance. The alpha parameter of the elastic net was fixed at 0.95 [20]. We counted the number of times each gene was selected by the elastic net over 100 bootstraps and subsequently ranked the genes by number of non-zero coefficients. We chose glmnet and plsDA as they are particularly suitable statistical methods for analysis of datasets with sparse numbers of observations and correlated variables, and provide accurate class estimates for the regression coefficients for selected genes and probability estimates for each sample. Multivariate analyses were performed in SPSS version 22 (SPSS Statistics, IBM, Chicago, IL) using linear regression to test the influence of age, sample collection site, sample source, and time post-transplant on the performance of kSORT with a level of significance set to *p*≤0.05. Receiver operating characteristic (ROC) curves were generated in GraphPadPrism vversion 5 (GraphPad, La Jolla, CA). For comparison of the areas under the different ROC curves, the method by Hanley and McNeil was applied with a threshold for significance set at *p*≤0.05 [23].

#### kSORT and kSAS development on ABI Viia7 data for prospective analysis of acute rejection

Given the underlying heterogeneity of the AART study samples, due to differences in sample handling and co-morbidities in the adult population versus the pediatric cohort, and to avoid the step of initial data normalization [24], a new correlation-based algorithm named kSAS was developed to allow for robust and easier classification of AR. kSAS was developed to apply fixed AR and No-AR QPCR reference profiles across the 17-gene panel to allow for accurate prospective prediction of samples independent of input sample number, and thus kSAS is feasible for translation to a routine clinical assay. kSAS uses QPCR dC_T_ or ddC_T_ values generated from two reference QPCR profiles (one for known AR and one for known No-AR), derived from the mean gene expression signature (centroid) obtained from multiple AR or No-AR samples. When a given sample gene expression profile is analyzed in kSAS, its expression is correlated to the reference profiles for one or more developed gene sets (models). In the Fluidigm dataset, kSAS was used to identify a single model composed of 14 of the 17 genes. For the ABI analyses, multiple equivalent top-performing gene sets (*n* = 13) were discovered from the analysis of samples from two collection sites (UCSF and UPMC) by randomly selecting and testing the performance of all possible permutations of different-sized gene sets of the 17 genes. Prior to discovery and testing of the ABI samples in the AART100 dataset, reference centroids were developed for different protocols (sample collection/processing methods). All ABI samples were evaluated against this database of reference centroids, with results reported for the centroid pair (AR and No-AR) with the greatest correlation to the evaluated sample (independent of collection methods). Classification of a sample as AR, No-AR, or indeterminate was derived by computing the Pearson correlation coefficients for all sample–centroid comparisons, assigning a score of 1 to samples with a greater correlation to an AR centroid and a score of −1 to samples with a greater correlation to a No-AR centroid. A kSAS aggregated AR risk score (−13 to 13) was then determined by summing the scores for all evaluated gene models. Indeterminate samples were defined as those with a score less than 9 and greater than −9, based on evaluation of sample classification. All gene models are provided in Table S1.

## Results

### Selection of 17 genes (kSORT) for Acute Rejection Classification and Prediction

**Figure 2:**
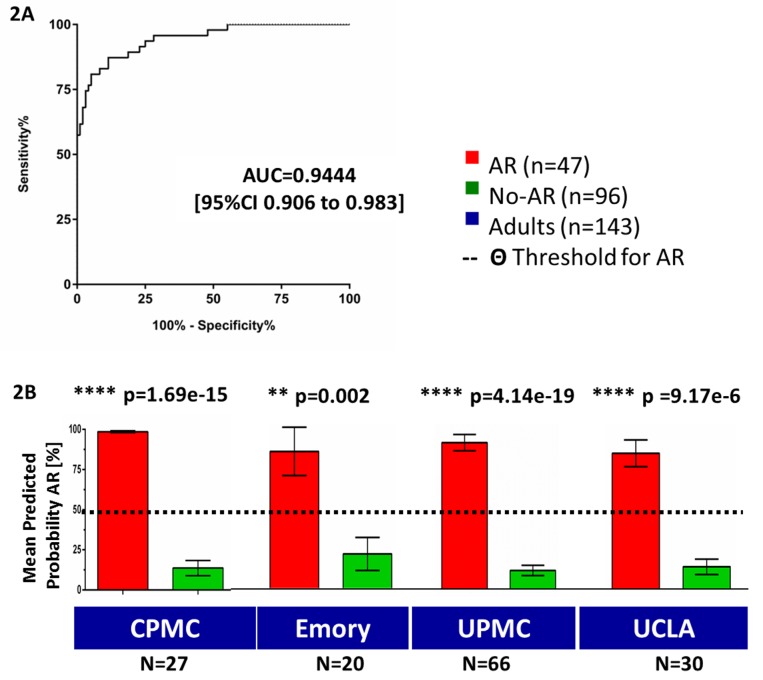
Training of a 17-gene model (kSORT) for acute rejection classification in 143 adult samples from real-life settings. 17 genes were used to classify 143 adult blood samples from four different sites into AR and No-AR by plsDA (threshold for AR prediction Θ was set at Θ = 50%). (A) AUC for AR in the training set was 0.94 (95% CI 0.91–0.98). (B) Predicted probabilities of AR were significantly higher in AR samples versus No-AR samples for each collection site and did not reach the threshold for AR prediction in the No-AR samples (predicted probability threshold Θ; Θ≥50%). Mean predicted probabilities for AR were 98.6%, 75.9%, 86.8%, and 77.2% for CPMC, Emory, UPMC, and UCLA, respectively. Mean predicted probabilities for No-AR were 13.7%, 19.4%, 11.3%, and 12.8% for CPMC, Emory, UPMC, and UCLA, respectively. Graphs show mean predicted AR probabilities in percent plus standard error of mean; *p*-values were calculated by two-sided Student's *t* test with Welch correction in case of unequal variances.

Initial evaluation of our previous ten-gene panel [2], which was highly predictive of pediatric AR in the prospective SNS01 clinical trial, was conducted in the AART143 training set of adult samples (collected by non-standardized non-clinical-trial protocols). Logistic regression modeling for these ten genes (*DUSP1, CFLAR, ITGAX, NAMPT, MAPK9, RNF130, IFNGR1, PSEN1, RYBP, NKTR*) correctly predicted 87.4% of the samples (specificity of 93.8% [95% CI 86.89%–97.67%], sensitivity of 74.50% [95% CI 59.65%–86.06%]), with an area under the ROC curve (AUC) of 0.86 (95% CI 0.79–0.93) (Figure S2). Out of the 43 genes tested, 31 genes were differentially expressed in AR in adult renal transplant recipients, and eight of these 31 differentially expressed genes (*q*-value≤0.05) were part of the original ten genes analyzed [2]. In the AART143 dataset, consisting of adult samples only, a subset of 15 of the 43 genes (*CEACAM4, CXCL10, GZB, IL2RB, RARA, RHEB, SLC25A37, C1orf38, EPOR, GZMK, ABTB1, NFE2, FOXP3, MMP1, MAP2K3*) correctly classified 91.6% of the AR and No-AR samples (elastic net classification with nested cross-validation) with a sensitivity of 85.9% and a specificity of 94.2%. However, this approach was unsuccessful in identification of AR in a purely pediatric data subset of the SNSO1 clinical trial [2],[5],[13], as only four of the 15 genes were selected (*EPOR, GZB, NFE2, SLC25A37*). To maintain the integrity of the assay to discriminate AR irrespective of recipient age, we retained the original panel of ten genes [2] (*DUSP1, CFLAR, ITGAX, NAMPT, MAPK9, RNF130, IFNGR1, PSEN1, RYBP, NKTR*). The inclusion of seven additional genes (*SLC25A37, CEACAM4, RARA, RXRA, EPOR, GZMK, RHEB*) optimized the performance of the gene set for discriminating AR in both adult and pediatric samples. These seven genes were identified by differential expression analyses for AR versus No-AR in both AART143 (adult samples only) and AART124 (adult and pediatric samples) (*q*-value≤5%: *CEACAM4, RARA, RXRA, SLC25A37*) and/or selected by the elastic net (*RHEB, EPOR, GZMK, CEACAM4, RARA, SLC25A37*) in the training set of AART143 samples. All 17 genes were selected (plsDA with equal prior probability) as the best gene set for discriminating AR in samples from adult patients (enrolled from four different US centers). The 17 genes predicted 39 of 47 AR samples correctly as AR, and 87 of 96 No-AR samples correctly as No-AR, resulting in a sensitivity of 82.98% (95% CI 69.19%–92.35%) and specificity of 90.63% (95% CI 82.95%–95.62%), with a calculated AUC of 0.94 (95% CI 0.91–0.98; *p*<0.001) (Figure 2A; Table 2), which was significantly increased compared to the AUC for the pediatric ten genes alone (one-sided *p* = 0.027, two-sided *p* = 0.054 [23]). Mean predicted AR probabilities were significantly different for AR versus No-AR in samples from each of the four individual centers included in the AART143 dataset (CPMC: AR, 98.60%±0.01; No-AR, 13.70%±0.24; *p*<0.001; Emory: AR, 76.00%±0.38; No-AR, 19.4%±0.3; *p* = 0.002; UPMC: AR, 86.80%±0.19; No-AR, 11.60%±0.21; *p*<0.001; UCLA: AR, 77.20%±0.35; No-AR, 11.40%±0.16; *p*<0.001) (Figure 2B). A flow diagram for the gene selection is provided in Figure S2.

**Table 2:** Performance of kSORT in the AART143, AART124, and AART100 cohorts.

Statistics	kSORT Predictions					
	AART143 (Training Set)		AART124 (Validation Set)		AART100 (Cross-Validation Set)	
	AR	No-AR	AR	No-AR	AR	No-AR
**Real Results**						
AR	39	8	21	2	36	3
No-AR	9	87	1	100	3	43
**Sensitivity (95% CI)**	82.98% (69.19%–92.35%)		91.30% (71.96%–98.93%)		92.31% (79.13%–98.38%)	
**Specificity (95% CI)**	90.63% (82.95%–95.62%)		99.01% (94.61%–99.97%)		93.48% (82.1%–98.63%)	
**PPV (95% CI)**	81.25% (68.06%–89.81%)		95.46% (78.20%–99.19%)		93.21% (79.68%–97.35%)	
**NPV (95% CI)**	91.58% (84.25%–95.67%)		98.04% (93.13%–99.46%)		93.48% (82.45%–97.76)	
**AUC (95% CI)**	0.94 (0.91–0.98)		0.95 (0.88–1.00)		0.92* (0.86–0.98)	

*AUC was calculated based on kSORT scores for *n* = 100 patients including high risk for AR (*n* = 39), low risk for AR (*n* = 46), and indeterminate (*n* = 15).

NPV, negative predictive value.

### Independent Validation of the 17 Genes (kSORT) in a Test Set of 124 Adult and Pediatric Recipients

**Figure 3:**
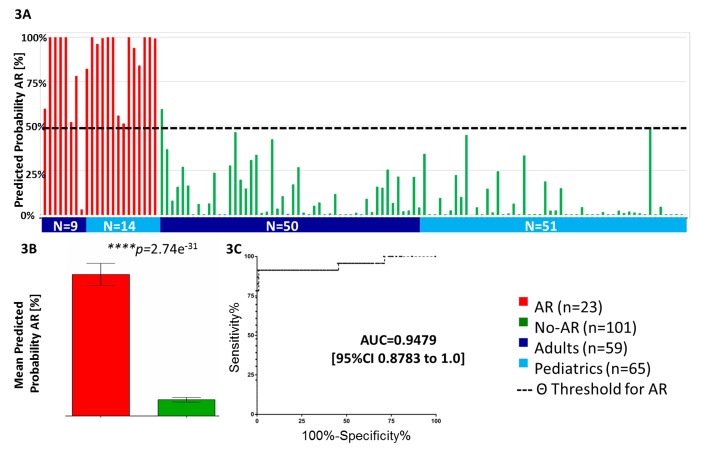
Validation of kSORT in 124 independent samples across different ages and settings. (A) Independent validation of kSORT in 124 adult and pediatric AR and No-AR blood samples using the fixed plsDA model on the Fluidigm platform. 22 out of 23 AR samples were correctly classified as AR (red bars), and 100 out of 101 No-AR samples were correctly classified as No-AR (green bars). Shown are individual predicted AR probabilities (percent) grouped by patient age (adult, ≥20 y; pediatric, <20 y) and phenotype. (B) predicted AR probabilities were significantly higher in the AR (80.6%) versus No-AR (9.2%) samples (*p*<0.001) included in the AART124 group (shown are mean predicted AR probabilities in percent with standard error of mean; two-sided Student's *t* test with Welch correction was applied to calculate *p*-values). (C) ROC analyses demonstrated high sensitivity and specificity for AR classification by the 17 genes.

To independently validate the 17-gene plsDA model developed in the AART143 samples (Fluidigm QPCR), we tested its performance in a combined adult (*n* = 59) and pediatric (*n* = 65) set of 124 independent samples (AART124). In the AART124 cohort, the 17-gene plsDA model predicted 21 of 23 AR samples correctly as AR, and 100 of 101 No-AR samples correctly as No-AR (Figure 3A; Table 2), including four patients with BKV nephritis, yielding an assay sensitivity of 91.30% (95% CI 71.96%–98.93%) and specificity of 99.01% (95% CI 94.61%–99.97%). One of the two misclassified AR samples had severe chronic damage (interstitial fibrosis/tubular atrophy grade III), with >33% global obsolescence in the biopsy sample at time of rejection. As seen in the training set (AART143), mean predicted probabilities of AR were also significantly different between the AR (80.55%±0.30) and No-AR (9.20%±0.13) samples (*p*<0.001; Figure 3B) in this validation set (AART124). Mean predicted probabilities of AR in the BKV group were low, at 12.76%. ROC analyses in the 124 samples resulted in an AUC of 0.95 (95% CI 0.88–1.00) (Figure 3C). Two patients in the AART124 cohort had an independent sample analyzed as part of the AART143 cohort.

### Equal Performance of kSORT in Samples from Four Different Sample Collection Sites

To evaluate the performance of the 17-gene plsDA model at each adult transplant center, we calculated AUCs for predictions at Emory (*n* = 42), UPMC (*n* = 81), UCLA (*n* = 44), and CPMC (*n* = 35) from AART143 and AART124. The performance of the assay was equivalent at each transplant center ([23]; *p*>0.05), with individual AUCs>0.8 at all four centers (Figure S3).

### Equal Detection of Antibody-Mediated and Cell-Mediated Acute Rejection

Most of the AR samples analyzed on the Fluidigm platform showed a mixed setting of some cellular and humoral rejection or associated chronic changes. No difference in AR prediction scores between 19 patients with clear antibody-mediated rejection (ABMR) only (C4d+ biopsy staining, DSA+) and 51 patients with clear cell-mediated rejection (TCMR) (C4d− and DSA−, and Banff t and i scores >1) was observed when assessed by the plsDA 17-gene model (*p* = 0.99 for TCMR versus ABMR; TCMR mean = 80.84%±4.40; ABMR mean = 80.75%±6.60; Figure S4).

### AR Prediction Is Independent of Time Post-Transplantation and Recipient and Donor Age

Multivariate analyses in the AART143, AART124, and AART100 cohorts evaluated the independence of kSORT performance from time post-transplantation and recipient and donor age. By linear regression, none of the tested variables except for the predicted probability of AR (kSORT score) significantly influenced the outcome for AR versus No-AR (*p* = 0.009). We additionally evaluated the performance of kSORT individually in the AR and in the No-AR groups for time post-transplantation categorized into 0–6 mo, 6 mo–1 y, and >1 y post-transplantation; comparing the kSORT scores using a one-way ANOVA model with Tukey's post-test for multiple comparisons, *p*-values in each group did not reach significance (AR, *p* = 0.22; No-AR, *p* = 0.60). Post-test for linear trend analysis in both the AR and No-AR groups confirmed no significant association with time post-transplantation (AR, *p* = 0.10; No-AR, 0.36) (Figure S4).

### Prediction of Biopsy-Confirmed Acute Rejection Prior to Clinical Graft Dysfunction

**Figure 4:**
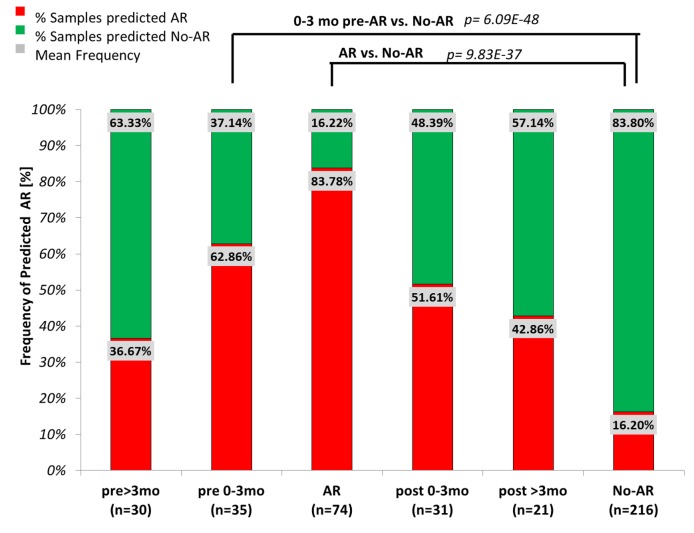
Evaluation of kSORT to predict acute rejection in 191 serially collected samples. 191 blood samples serially collected within 6 mo before (pre) or after (post) biopsy-confirmed AR were evaluated by kSORT. Frequencies of samples predicted as AR (red) or predicted as No-AR (green) were compared between sample collection time points (>3 mo prior to AR biopsy, *n* = 30; 0–3 mo prior to AR biopsy, *n* = 35; at AR biopsy, *n* = 74; 0–3 mo after AR biopsy, *n* = 31; >3 mo after AR biopsy, *n* = 21; and at No-AR/stable time points, *n* = 216). 62.86% of samples collected 0–3 mo (1.15±0.90 mo) prior to the AR biopsy had high probabilities for AR predicted by kSORT (96.36±0.08). High probabilities for AR persisted in 51.6% of samples collected 0–3 mo post-AR (94.60%±0.14); in comparison, 83.8% of the No-AR samples were always predicted as No-AR (8.20%±0.12). Mean AR scores were significantly different between pre-AR samples (0–3 mo) and No-AR/stable samples, as well as between AR samples and No-AR/stable samples.

To evaluate the predictive nature of the 17-gene plsDA model, 191 blood samples (AART191; prospective validation) drawn either before (0.2–6.8 mo, *n* = 65) or after (0.2–7 mo; *n* = 52) a biopsy-matched AR episode (*n* = 74) were analyzed. Out of the patients with blood samples 0–3 mo prior to the AR biopsy (*n* = 35), at time of stable graft function, 62.9% (22 out of 35 samples) had very high AR prediction scores (96.4%±0.8) (Figure 4; Table 3), significantly greater than scores in patients with stable graft function and no AR on follow-up (19.4%±0.3; *p*<0.001). Of the patients with blood samples drawn 0–3 mo after AR treatment (*n* = 31), 51.6% (16 out of 31) still had elevated predicted AR scores (86.00%±0.17); the remaining 15 samples showed AR scores below the threshold θ for AR (θ = 50%; 6.59%±0.13%) at 0–3 mo after AR treatment. As serum creatinine levels in patients with elevated AR prediction scores were 2.04±0.40 mg/dl compared to creatinine levels of 1.8±0.4 mg/dl in patients with decreased AR prediction scores, the latter likely represented patients who responded to AR treatment (Figure 4).

**Table 3:** kSORT evaluation in AART191 for AR prediction.

	Time from Rejection*	Percent Samples Predicted as AR	Predicted AR Probability**	Percent Samples Predicted as No-AR	Predicted AR Probability**
**Pre >3 mo**	−6.38±5.20	36.67%	95.37%±0.10	63.33%	13.44%±0.20
**Pre 0–3 mo**	−1.15±0.90	62.86%	96.36%±0.08	37.14%	11.71%±0.20
**AR**		83.78%	93.20%±0.10	16.22%	26.36%±0.20
**Post 0–3 mo**	1.25±0.80	51.61%	85.96%±0.20	48.39%	6.59%±0.10
**Post >3 mo**	5.57±2.70	42.86%	94.63%±0.10	57.14%	1.48%±0.03
**No-AR**		16.20%	80.30%±0.20	83.80%	7.48%±0.10

*Mean time from rejection in months ± standard deviation.

**Mean predicted probability of AR ± standard deviation.

### The Biological Basis of the 17 Genes

When evaluated in the context of experimentally predicted interactions, more than half of the 17 genes in the plsDA model were directly or indirectly associated with each other by common molecular pathways (Figure S5), particularly regulation of apoptosis, immune phenotype, and cell surface. In addition to the ten genes previously evaluated as peripheral biomarkers for pediatric AR, and known to be mostly expressed in PB cells of the monocyte lineage [2], six of the additional seven peripheral AR genes [25] were also expressed by activated monocytes (*RXRA, RARA, CEACAM4*), endothelial cells (*EPOR, SLC25A37*), and T cells (*GZMK*) in the peripheral circulation. Eleven of the 17 genes played a common role in cell death and the cell survival network (Fisher's exact test, *p*<0.05; Ingenuity Pathway Analysis, [Qiagen, Valencia, CA]; Figure S5).

### Locking the 17-Gene kSORT Assay on the ABI Viia7 QPCR Platform with kSAS, a Customized Analysis Suite

**Figure 5:**
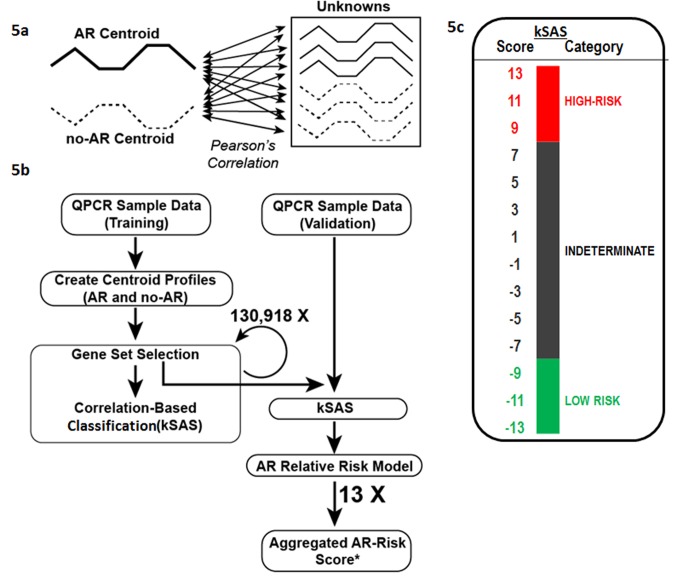
Development of kSAS and the kSORT assay. kSAS was developed to provide individual sample AR risk scores and AR risk categories. (A) Expression values of the 17-gene kSORT model in unknown samples were correlated to corresponding AR and No-AR reference values (centroids) by Pearson correlation. (B) For kSORT assay development, QPCR data from 100 samples were divided into training (*n* = 32) and independent validation sets (*n* = 68). (C) 13 12-gene models from the 17-gene kSORT model generated numerically aggregated AR risk scores for each sample and categorized them into three groups: high risk for AR (aggregated AR risk score ≥9), low risk for AR (aggregated AR risk score ≤−9), and indeterminate (aggregated AR risk score <9 and >−9) category.

Having established the performance of the kSORT plsDA model for detection and prediction of biopsy-confirmed AR in pediatric and adult patients, the final kSORT assay was developed on the ABI platform (ABI Viia7). To mitigate the data normalization required to control for batch effect and sample handling differences, we developed a new algorithm called kSAS (Figure 5A) to classify samples using multiple covariate-impacted reference sets, based on expression correlation (Pearson) of an individual sample to each reference profile of AR and No-AR. Rather than relying on a single gene set, this method creates and uses multiple gene subsets (models), each of which is then correlated to a reference AR or No-AR expression profile for a given sample to provide a combined model score for each reference. Samples with multiple disagreeing model predictions (*n*>2) are considered indeterminate and thus cannot be confidently assigned as AR or No-AR. The kSORT kSAS assay was developed and cross-validated in 100 independent adult and pediatric samples collected from 44 patients with AR and 56 patients without AR (Figure 5A).

#### Evaluation of kSAS performance in the Fluidigm dataset

To develop the kSAS algorithm, we utilized the Fluidigm QPCR dataset of the AART143 cohort and split the samples into a training set of *n* = 95 and a test set of *n* = 48 by random selection. From the 17 AR genes evaluated, kSAS identified a single gene set model composed of 14 genes (*CEACAM4, CFLAR, DUSP1, EPOR, IFNGR1, NAMPT, NKTR, PSEN1, RARA, RHEB, RNF130, RXRA, RYBP, SLC25A37*) representing a combination of eight of the ten genes from the Li et al. study [2] and six of the seven genes from the new selection.

#### Locking the kSAS score in the ABI dataset

To account for possible sample-protocol-associated covariates, mean gene expression measurements (centroids) were calculated from known AR and known No-AR samples for the major experimental covariates (sample collection/processing methods) to create a panel of possible reference AR and No-AR profiles. For each evaluated sample, kSAS automatically selects the most correlated reference set for downstream classification. In contrast to other classification algorithms, kSAS does not apply fixed mathematical coefficients, and thus its output is not affected by differences in RNA preparation and handling variances, which can affect QPCR data ranges. Blood samples (*n* = 32, UCSF and UPMC) confirmed as AR (*n* = 16) or No-AR (*n* = 16) were evaluated by kSAS and tested for the performance of over 130,000 different possible gene combinations (3–17 genes per model). This analysis yielded 13 different 12-gene combinations utilizing a total of 15 of the 17 genes (*CEACAM4, CFLAR, DUSP1, EPOR, GZMK, ITGAX, MAPK9, NAMPT, NKTR, PSEN1, RARA, RHEB, RXRA, RYBP, SLC25A37*), with seven core genes present in all 13 12-gene combinations (*CEACAM4, CFLAR, EPOR, GZMK, NKTR, PSEN1, SLC25A37*) (Table S1). As our multiple model approach provided distinct scores for each gene set, we evaluated the combined ability of these models to provide a single confidence score for each patient that is not based on a single gene model but includes all 13 12-gene models. This aggregated AR risk analysis produced a numerical AR risk score for each patient (−13 to 13), by simply subtracting the number of times a patient was predicted as No-AR by the 13 12-gene models from the number of times the same patient was predicted as AR.

#### Validation of kSAS in independent samples from adult and pediatric transplant recipients

**Figure 6:**
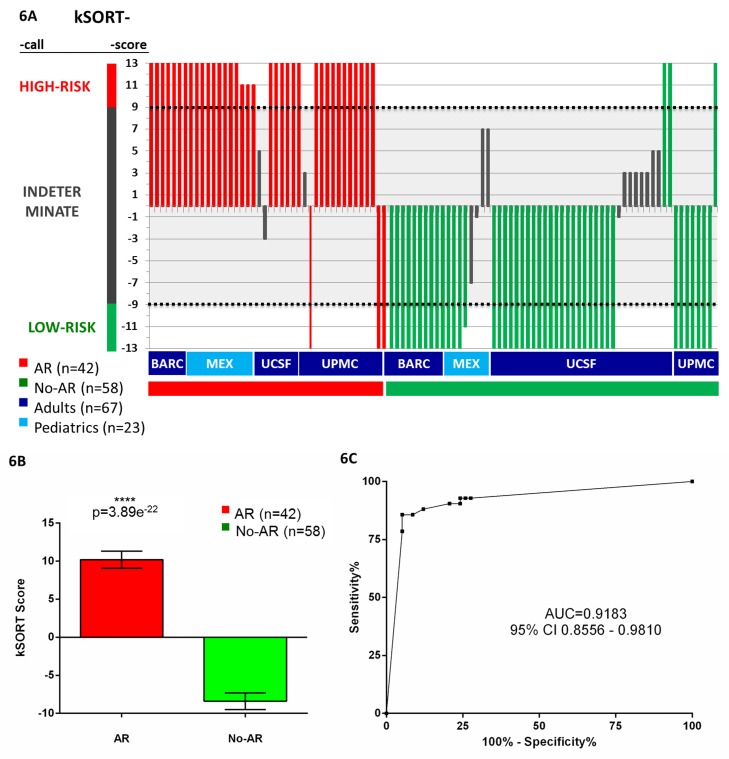
Performance of the kSORT assay. (A) kSORT score and classification category were calculated for each sample: the kSORT assay correctly classified 36 out of 39 AR samples as high risk for AR (red bars, 92.3%; risk score ≥9) and 43 out of 46 No-AR samples as low risk for AR (green bars, 93.5%, risk score ≤−9) across four different sample collection sites, and adult (≥20 y) versus pediatric (<20 y) recipient age groups, with samples collected in clinical trial and non-clinical-trial settings; remaining 15 samples classified as indeterminate risk for AR (grey bars; risk score <9 and >−9). (B) Mean aggregated kSORT scores (error bars give standard error of mean) were significantly higher in all true AR samples (10.2±7.2) than in all true No-AR samples (−8.4%±8.2) in the AART100 cohort by two-sided Student's *t* test. (C) ROC analysis demonstrated high sensitivity and specificity for the kSORT assay in the AART100 cohort.

To assess the performance of the fixed kSAS algorithm utilizing the 13 12-gene models, we calculated aggregated AR risk scores for the combined dataset of 100 AR and No-AR samples (26 AR, 42 No-AR; AART100). The large majority of samples were classified as AR or No-AR with all kSAS gene models in agreement (81 out of 100). Given the large agreement in model predictions, to minimize false positive AR predictions, if three or more disagreeing calls (∼1/4 of the models) occurred, the sample was classified as indeterminate, otherwise the sample was predicted to be high risk for AR (≥11 AR calls; combined score ≥9), or low risk for AR (≥11 No-AR calls; combined score ≤−9) (Figure 5). Among patients predicted as high risk, 92.3% were classified correctly as AR (36 out of 39); among patients predicted as low risk for AR, 93.5% (43 out of 46) were classified correctly as No-AR based on the biopsy reading (Table 2), resulting in a sensitivity of 92.31% (95% CI 79.13%–98.38%) and specificity of 93.48% (95% CI 82.10%–98.63%). The remaining patients (*n* = 15) were predicted as indeterminate (Figure 6A). The combined kSORT scores in the AR samples (10.2±7.2; *n* = 42) were significantly higher than those in the No-AR samples (−8.4%±8.2; *n* = 58) by two-sided Student's *t* test, with *p*<0.001 (Figure 6B). The AUC for the combined kSORT scores from all 100 samples was 0.918 (95% CI 0.856–0.981) (Figure 6C).

## Discussion

Clinical availability of molecular tests for accurate disease diagnosis and patient stratification for treatment regimens is a pressing issue in medicine. Molecular classification of acute transplant rejection independent of the invasive biopsy procedure is a much needed clinical assay [10],[26]. Recent scientific research has highlighted the deficiencies of using histology alone as a means for detecting AR, with its inherent problems of sampling, read variability, and the inability to use the biopsy for serial monitoring and AR prediction [7],[8].

In the present study we developed a simple yet sensitive and specific noninvasive QPCR test, kSORT, together with a novel algorithm, kSAS, for the detection and prediction of acute renal allograft rejection from a PB sample, irrespective of the rejection grade or pathological diagnosis of antibody- or cell-mediated rejection. kSORT accurately indicated the risk and prevalence of acute renal rejection in 558 PB samples from 436 adult and pediatric renal transplant patients based on the expression of 17 genes (final AUC = 0.92, 95% CI 0.86–0.98). The kSORT assay performance was independent of age, time post- transplantation, transplant-specific patient management, and sample processing. The kSAS algorithm, which correlates the expression of an individual patient sample to multiple gene AR and No-AR reference profiles across multiple overlapping gene models, was successful at classifying samples with known sample handling variables.

The approach we applied here combined our previous findings in pediatric transplant patients in a clinical trial setting with a novel discovery in adult and pediatric transplant patients from a “real-life” setting. We advanced our recent transcriptional panel of ten genes previously shown to be differentially regulated in blood from patients with AR in different solid organs (the kidney [2],[26] and the heart [4],[27]). This initial AR panel assay was developed in a cohort of pediatric-only renal transplant patients with low risk of AR (pre-transplant panel reactive antibody level <20%; *n* = 279; ages 1–21 y) as part of an NIH-funded randomized trial in 12 US centers [26], with stringent control of the standard operating procedure for patient care and blood sample processing. We performed additional QPCR in PB samples collected in the present AART study, which represents a real-life heterogeneous transplant cohort of 436 adult and pediatric patients from eight independent transplant centers, where samples were collected irrespective of the patients' immunologic risk, immunosuppression, and co-morbidities, and where different blood RNA sampling methodologies were applied. This approach allowed for the development of an assay for which performance is robust against clinical and experimental confounders—important assets of kSORT and the kSAS algorithm, as multiple studies have shown that such confounders are associated with wide variability in the quality and reproducibility of gene expression data [28]–[31] and are likely to confound the interpretability of test results.

Another strength of the kSORT assay is its high positive predictive value (PPV) (93.21%) for detecting AR in a PB sample. The only PB diagnostic test that is currently available for detecting transplant rejection in transplant patients [32] detects the absence of moderate/severe acute cellular cardiac rejection (ISHLT 3A), but provides low sensitivity for detection of the presence of AR (PPV = 6.8%) [32]. Similarly, a blood gene expression test for assessing obstructive coronary artery disease (CorusCad, CardioDx, Palo Alto, CA) yielded a PPV of 46% in a multicenter validation study [33]. In addition to the high sensitivity of kSORT to detect AR at the point of rejection (as diagnosed by the current gold standard), kSORT was able to identify subclinical AR in 12 out of 16 subclinical rejections that were included in the AART100 cohort and predicted clinical AR in >60% of samples collected up to 3 mo prior to graft dysfunction and histological AR—an important ability for a rejection test, as subclinical and clinical AR are precursors of chronic rejection and graft loss [5]. Although current immune-monitoring tools for assessing the adaptive alloimmune response have shown their usefulness for predicting the potential risk of AR [34],[35], detection of donor-specific antibodies or memory-effector T cells does not necessarily translate to ongoing immune-mediated allograft damage, and these effector mechanisms may not always be detected prior to or at the time of biopsy-proven AR. Furthermore, most centers currently do not perform protocol biopsies as a means to detect subclinical AR, and thus subclinical cases remain largely undetected. The kSORT assay is promising for routine noninvasive post-transplant monitoring to predict AR and limit graft damage with timely intervention. The high specificity of the kSORT assay suggests its potential value to be additionally used as a negative predictor of rejection in the graft.

A constraint of the kSORT assay in its current assay design is its inability to discriminate between TCMR and ABMR in our study. Two potential explanations could account for this observation; on the one hand, a likely link lies in a common finding of monocyte activation in both types of AR, as the kSORT genes were highly enriched in monocytes. Indeed, monocytes are critical cells that can directly mediate the injury of tubulitis in TCMR in the allograft; additional evidence exists that donor-specific HLA class I antibodies in ABMR activate endothelial cells, which in turn can recruit and activate monocytes and leukocytes, increasing graft inflammation [36]. On the other hand, the common kSORT gene expression in both cellular and humoral rejections may represent hierarchical architecture of the adaptive immune response that takes place during the rejection process, rather than clustering with any specific effector mechanism responsible for each type of allograft rejection. In fact, some genes were also highly expressed in CD4+ and CD8+ T cells, whereas some others were highly up-regulated in B cells or dendritic cells, thus suggesting the close interplay between both main effector mechanisms of the adaptive immune response during allograft rejection [37]. Recent studies where gene expression analyses allowed for discrimination of ABMR and TCMR were based on transcriptional studies of the rejecting biopsy tissue [38], most valuable to better understand underlying molecular mechanisms that may differentiate ABMR and TCMR based on injury localization in specific compartments of the kidney biopsy (endothelial cells in ABMR versus tubular cells in TCMR) or the specific major effector mechanisms of injury (natural killer cells in ABMR versus cytotoxic T cells in TCMR) [2],[4],[39]–[42]. Of note, down-regulation of kSORT genes in patients with other underlying inflammatory processes such as BKV infection and non-allospecific nephritis suggests that despite being a monocyte-driven transcriptional signature, kSORT illustrates the activation of an allogeneic immune response.

Similar to our recent observation of a common rejection module (CRM; *n* = 11 genes) in tissue across heart, liver, lung, and kidney allografts [3], the present study in blood also finds genomic commonalities for the 17 genes across kidney [2] and heart [4] and across pediatric [2] and adult [4] transplant recipients. The combined learning in blood and tissue supports the postulation that immune perturbation is global, synonymous across transplant organs and unaffected by age, suggesting a common immunological axis. This apparent commonality supports the future evaluation of kSORT for patient monitoring beyond kidney transplantation. Nevertheless, the CRM genes detected in tissue do not overlap with our kSORT genes detected in blood, which is not surprising as the predominant expression changes in the graft reflect cellular infiltration and inflammation, and are likely different from the signals of activated trafficking cells in the periphery.

A limitation of our study is the currently unavoidable fact that the reference for evaluation of our blood test performance is based on histological readings, with their aforementioned shortcomings, which serve as the current gold standard for detection of rejection. Moreover, the lack of centralized histological information for all biopsy samples might be considered as an additional constraint, even though all tissue samples were evaluated following the current gold standard Banff score classification for the description of main allograft lesions [14]. Furthermore, kSORT needs to be evaluated in a prospective clinical trial before its clinical efficacy can be determined. To clinically evaluate the diagnostic and predictive performance of kSORT, we have included patient monitoring by kSORT in a prospective, randomized, double-blinded clinical trial (Steroid Avoidance and Low-Dose CNI by ATG-Induction in Renal Transplantation [SAILOR]; EU Clinical Trials Register SUGBG-012011). The results of this trial will further evaluate whether kSORT can be used as an objective and quantitative measure for the risk of AR, and whether kSORT can be used serially post-transplant to complement current clinical practice guidelines for stratifying patient immune risk, medication load, and requirement for biopsy.

In conclusion, in this report we present the validation of the kSORT assay to detect risk of AR in renal transplant patients; the kSORT assay has the potential to become a simple, robust, and clinically applicable blood test.

## Supporting Information

Figure S1 Confounder analysis.
(DOCX)

Figure S2 Discovery of kSORT.
(DOCX)

Figure S3 Performance of kSORT by transplant center.
(DOCX)

Figure S4 kSORT detects antibody- and cell-mediated acute rejection; kSORT is independent of time post-transplantation.
(DOCX)

Figure S5 The biological basis of the 17 genes.
(DOCX)

Table S1 kSORT performance.
(DOCX)

Table S2 The exact number of samples and patients used from each of the participating centers in the AART study.
(DOCX)

Checklist S1 STARD checklist.
(DOCX)

## Abbreviations

AART: Assessment of Acute Rejection in Renal Transplantation
ABI: Applied Biosystems
ABMR: antibody-mediated rejection
ANOVA: analysis of variance
AR: acute rejection
AUC: area under the receiver operating characteristic curve
Barcelona: Bellvitge University Hospital
BKV: BK polyomavirus
CNI: calcineurin inhibitor
CPMC: California Pacific Medical Center
CRM: common rejection module
DSA: donor-specific antibodies
Emory: Emory University
kSAS: kSORT analysis suite
kSORT: Kidney Solid Organ Response Test
Mex: Hospital Infantil de México Federico Gómez Laboratorio de Investigacion en Nefrologia
NIH: National Institutes of Health
PB: peripheral blood
PBL: peripheral blood lymphocyte
PBMC: peripheral blood mononuclear cell
plsDA: partial least squares discriminant analysis
PPV: positive predictive value
QPCR: quantitative real-time PCR
ROC: receiver operating characteristic
Stanford: Stanford University
TCMR: cell-mediated rejection
UCLA: University of California Los Angeles
UCSF: University of California San Francisco
UPMC: University of Pittsburgh Medical Center
