# Correction: The kSORT Assay to Detect Renal Transplant Patients at High Risk for Acute Rejection: Results of the Multicenter AART Study

**DOI:** 10.1371/journal.pmed.1001790

**Published:** 2015-02-20

**Authors:** The PLOS Medicine Staff

The names of the eighth and nineteenth authors are incorrect. The correct name for the eighth author is: Adriana Zeevi. The correct name for the nineteenth author is: Elaine F. Reed.
