# Supplementary material for: The kSORT Assay to Detect Renal Transplant Patients at High Risk for Acute Rejection: Results of the Multicenter AART Study
Source: PLoS Med. 2014 Nov 11;11(11):e1001759. doi: 10.1371/journal.pmed.1001759 (PMC4227654; doi:10.1371/journal.pmed.1001759)

**Supporting Figure S1:**

**Confounder Analysis**

Principal component analysis (PCA) of QPCR data from 143 AR and No-AR adult samples (Cohort 1) for 43 rejection genes revealed sample segregation by sample collection site (1A) rather than phenotype (1B). Normalization of QPCR data by mixed ANOVA corrected for the dominant effect of sample collection site on gene expression (1C) and resulted in segregation of samples into AR and No-AR (1D). PCA was performed using relative gene expression values (dCt 18S) for 43 genes. A mixed ANOVA model was built with sample collection site, RNA source and chip as random categorical factors and phenotype as categorical factor. Each sphere represents a sample; colors in 1A and 1C reflect sample collection sites (green=UPMC; purple=UCLA; red=CPMC; blue=Emory); colors in 1B and 1C reflect patient phenotype (red=AR; blue=No-AR) based on biopsy diagnosis.


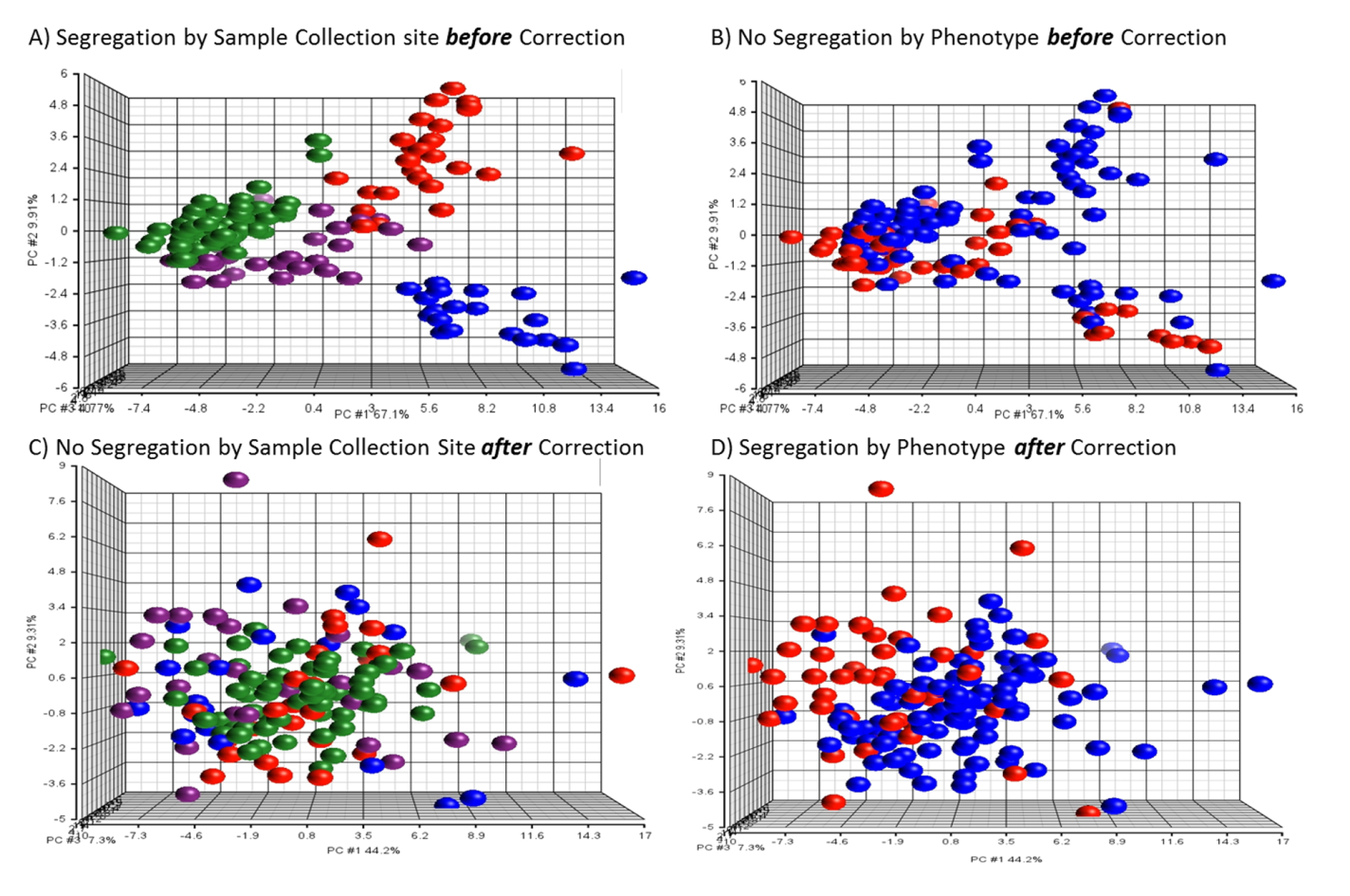

Supplement: Figure S1 — Confounder analysis. (DOCX) [file pmed.1001759.s001.docx]
