# Supplementary material for: The kSORT Assay to Detect Renal Transplant Patients at High Risk for Acute Rejection: Results of the Multicenter AART Study
Source: PLoS Med. 2014 Nov 11;11(11):e1001759. doi: 10.1371/journal.pmed.1001759 (PMC4227654; doi:10.1371/journal.pmed.1001759)
Supplement: Figure S2 — Discovery of kSORT. (DOCX) [file pmed.1001759.s002.docx]

**Supporting Figure S2: Discovery of kSORT**

Discovery of the final 17 AR genes for kSORT was done in gene expression data from 267 adult and pediatric blood samples (Cohort 1 AART143, Cohort 2 AART124) from the microfluidic high throughput Fluidigm QPCR performed for a total of 43 genes: 10 pediatric AR genes previously identified by us [[1](#_ENREF_1)]; 33 candidate genes for novel discovery in adult and pediatric transplant rejection. Confirming the pediatric 10 genes in the adult set of 143 AR and No-AR samples correctly predicted AR with 87.4%. Novel discovery and validation was performed in the combined adult and pediatric data set of 267 AR and No-AR samples (Cohort 1, Cohort 2). Student T-test, ANOVA and penalized logistic regression resulting in the definition of 7 additional genes which together with the 10 rejection set defined the final selection of 17 genes for kSORT. By partial least square discriminant analysis with equal prior probabilities the 17 genes predicted AR with high sensitivity and specificity in the training set of 143 Samples (Cohort 1; AART143, AUC=0.944) as well as in the independent Validation set of 124 samples (Cohort 2; AART124) not included in any previous analysis (AUC=0.948). Gene expression data used in the analysis represented dCt values against 18S from the Fluidigm QPCR platform additionally normalized for sample collection site, RNA source, and run using a mixed ANOVA model.


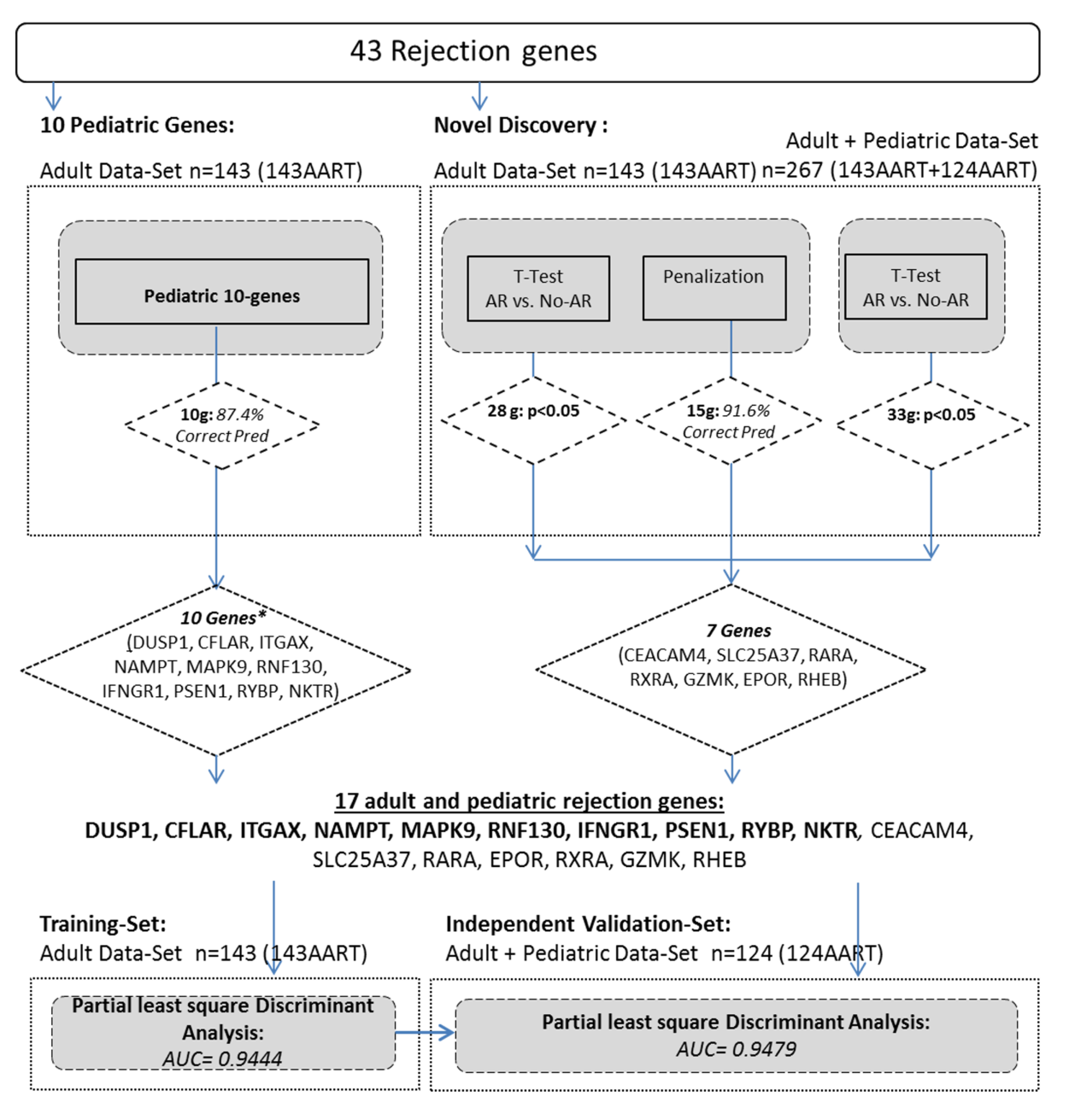


**References**

1. Li, L., et al., *A peripheral blood diagnostic test for acute rejection in renal transplantation.* Am J Transplant, 2012. **12**(10): p. 2710-8.
