# Supplementary material for: The kSORT Assay to Detect Renal Transplant Patients at High Risk for Acute Rejection: Results of the Multicenter AART Study
Source: PLoS Med. 2014 Nov 11;11(11):e1001759. doi: 10.1371/journal.pmed.1001759 (PMC4227654; doi:10.1371/journal.pmed.1001759)

**Supporting Figure S3: Performance of kSORT by Transplant Center**

ROC analyses were performed for each transplant center included in the AART study to assess the performance of kSORT across different sample collection sites. Calculated AUCs were 0.8765 (95%CI 0.7538 to 0.9993) for AR vs. No-AR collected at Emory University (4a; n=42); 0.9825 (95%CI 0.9608 to 1.0) for AR vs. No-AR collected at UPMC (4b; n=81), 0.9360 (95%CI 0.8648 to 1.0) for AR vs. No-AR collected at UCLA (4c, n=44), and 1.0 (95%CI 1.0 to 1.0) for AR vs. No-AR collected at CPMC (4d,n=35). The latter is an imbalanced data-set with only 2 AR samples and kSORT performance likely overfitted. Tables next to each ROC curve displays 2x2 tables for the kSORT performance in each center.


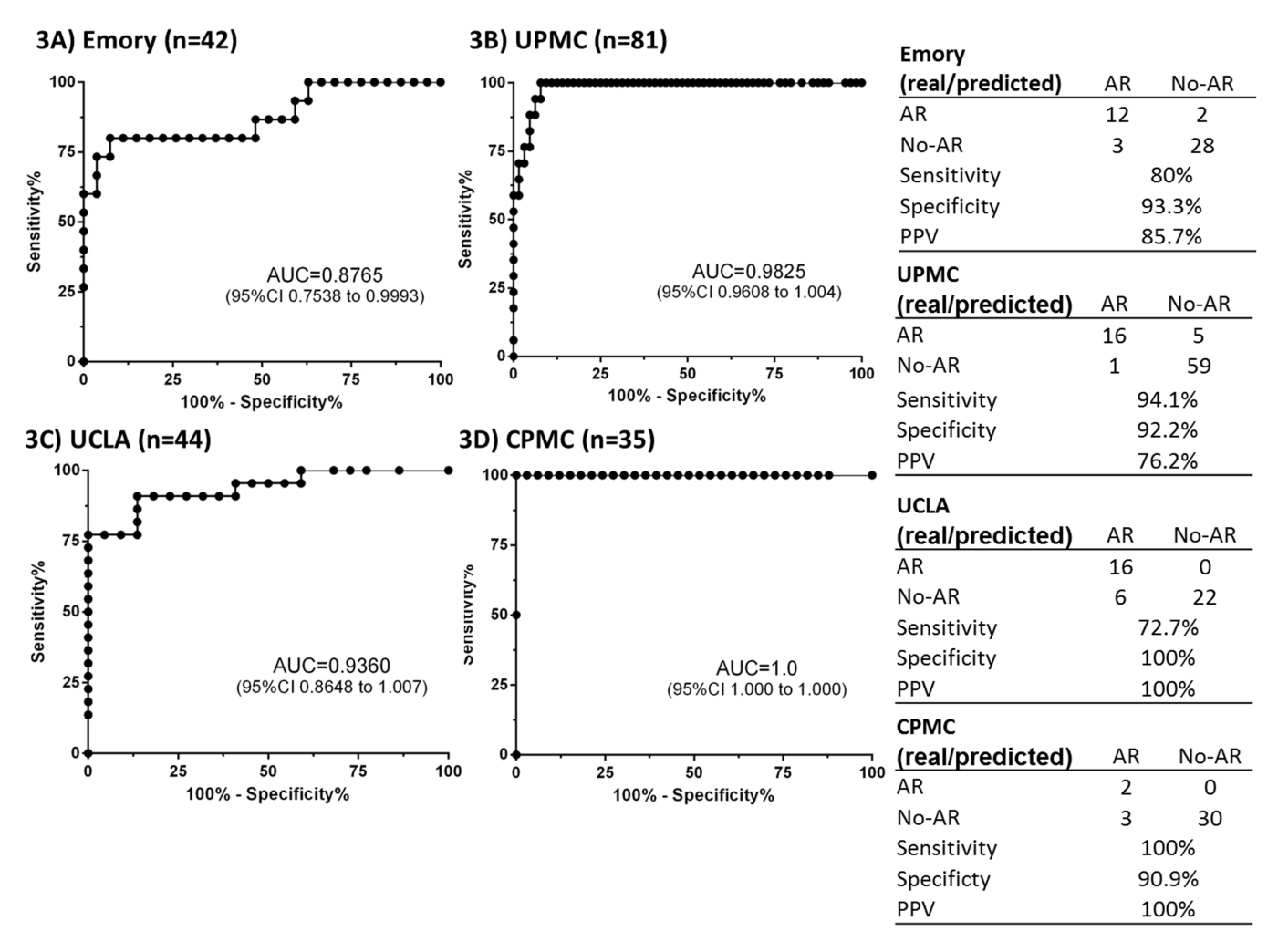

Supplement: Figure S3 — Performance of kSORT by transplant center. (DOCX) [file pmed.1001759.s003.docx]
