# Supplementary material for: The kSORT Assay to Detect Renal Transplant Patients at High Risk for Acute Rejection: Results of the Multicenter AART Study
Source: PLoS Med. 2014 Nov 11;11(11):e1001759. doi: 10.1371/journal.pmed.1001759 (PMC4227654; doi:10.1371/journal.pmed.1001759)

**Supporting Figure S4: kSORT detects antibody and cellular mediated acute rejection; kSORT is independent of time post transplantation**

Predicted probabilities of AR by the fixed 17 gene model was compared in a subset of 19 patients with clear antibody mediated rejection only (ABMR, C4D positive biopsy staining, DSA+) to a subset of 51 patients with clean cellular mediated rejection (TCMR, C4d- and DSA-); the fixed 17 gene model equally detected humoral and cellular AR (4A, plsDA, p=0.99; mean predicted AR-Probability TCMR=80.84%±4.4; ABMR=80.75%±6.6).

Similarly the 17 fixed gene plsDA model predicted AR independent of time post transplantation with continuous low predicted probabilities for AR in the No-AR patients and continuous high AR predicted probabilities in the AR patient group (S4b; mean predicted probability of AR plus SEM). Mean AR predicted probabilities were calculated for samples falling in 1 of 3 time post transplantation categories (0-6 months, 6months – 1 year, >1 year) and compared by Student T-test; p-values for significance analyses by ANOVA and linear trend did not reach significance (p>0.05).


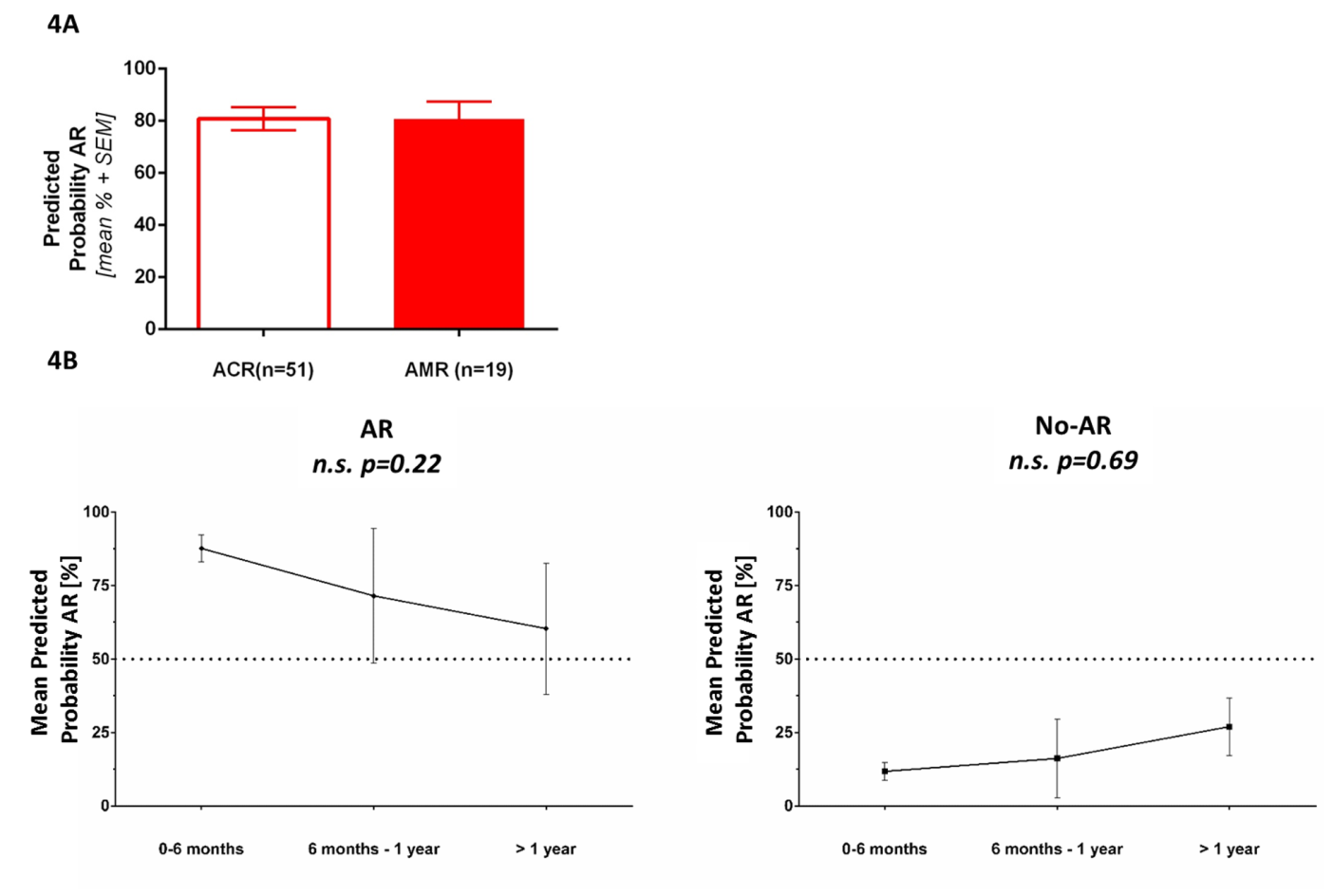

Supplement: Figure S4 — kSORT detects antibody- and cell-mediated acute rejection; kSORT is independent of time post-transplantation. (DOCX) [file pmed.1001759.s004.docx]
