# Supplementary material for: The kSORT Assay to Detect Renal Transplant Patients at High Risk for Acute Rejection: Results of the Multicenter AART Study
Source: PLoS Med. 2014 Nov 11;11(11):e1001759. doi: 10.1371/journal.pmed.1001759 (PMC4227654; doi:10.1371/journal.pmed.1001759)

**Supporting Figure S5: The biological basis of the 17 genes**

Pathway and Network analyses demonstrated strong biological correlation of genes supporting correlation seen in gene expression across AR and No-AR samples by QPCR. Significantly (p<0.05) associated with the 17 genes were regulation of apoptosis, immune phenotype and cell surface proteins (5A); Ingenuity Pathway Analyses (IPA, Qiagen, Redwood City, CA) further demonstrated a common role of 11 of the 17 genes in cancer, cell death and cell survival (p<0.05, 5B). Additional network analyses showed that 7 of the 17 genes formed a single network of direct interactions (5C).


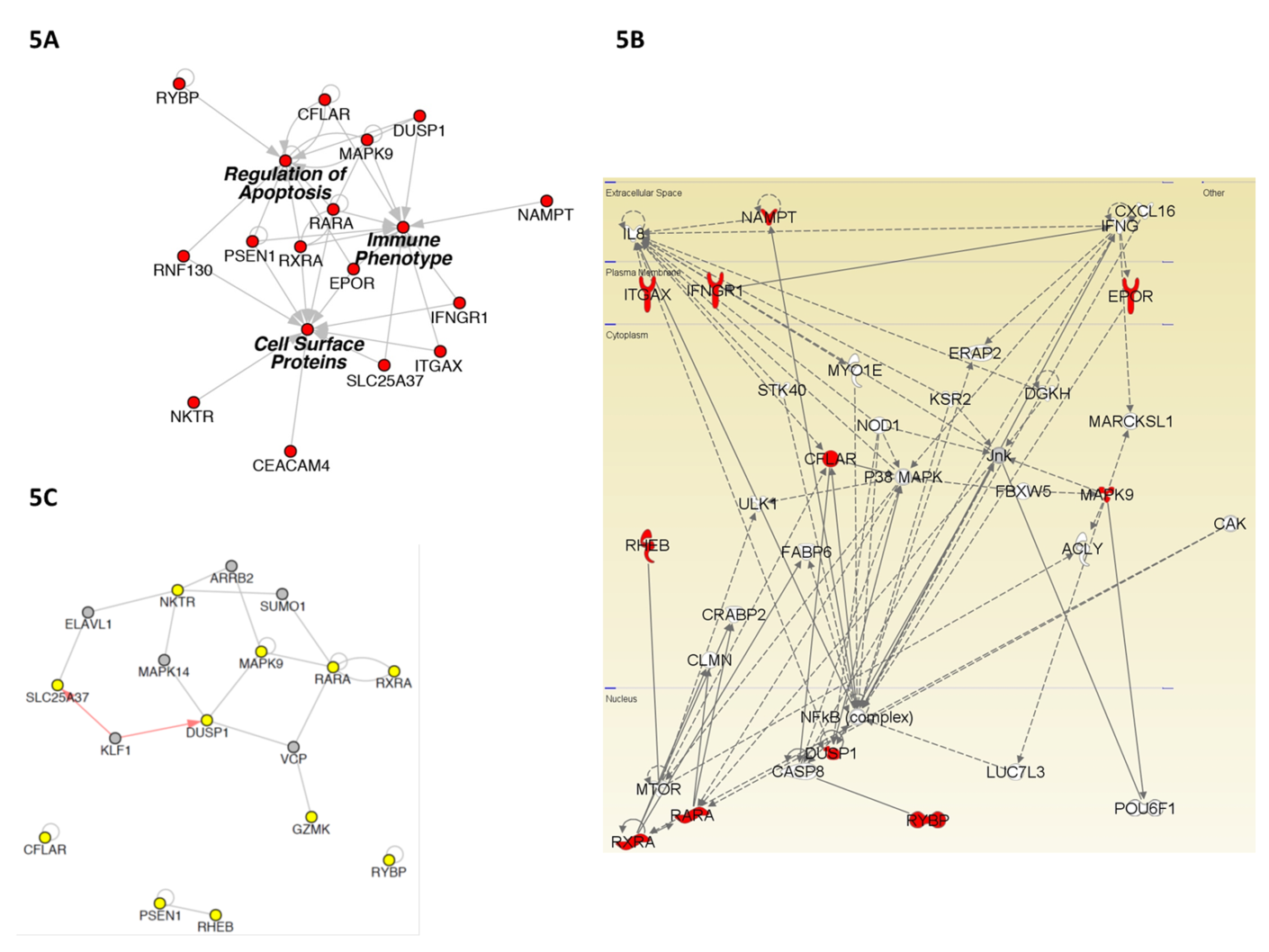

Supplement: Figure S5 — The biological basis of the 17 genes. (DOCX) [file pmed.1001759.s005.docx]
