# Supplementary material for: The kSORT Assay to Detect Renal Transplant Patients at High Risk for Acute Rejection: Results of the Multicenter AART Study
Source: PLoS Med. 2014 Nov 11;11(11):e1001759. doi: 10.1371/journal.pmed.1001759 (PMC4227654; doi:10.1371/journal.pmed.1001759)
Supplement: Table S1 — kSORT performance. (DOCX) [file pmed.1001759.s006.docx]

**Supporting Table S1: kSORT Performance (selected top 13 12-gene Models from selected 17 genes)**

| Model | Adult Training-Set (n=32) | Pediatric + Adult Test-Set (n=68) | Description |  |  |  |  | |  |  |  |
| --- | --- | --- | --- | --- | --- | --- | --- | --- | --- | --- | --- |
| **1** | **90.63%** | **88.24%** | **CFLAR, PSEN1, CEACAM4, NAMPT, RHEB, GZMK, NKTR, DUSP1, RARA, ITGAX, SLC25A37, EPOR,** | | | | |  |  |  |  |
| 2 | 90.63% | 86.27% | CFLAR, PSEN1, CEACAM4, NAMPT, RHEB, GZMK, NKTR, DUSP1, ITGAX, SLC25A37, RXRA, EPOR, | | | | |  |  |  |  |
| 3 | 90.63% | 86.27% | CFLAR, PSEN1, CEACAM4, RHEB, GZMK, NKTR, DUSP1, RARA, ITGAX, SLC25A37, RXRA, EPOR, | | | | |  |  |  |  |
| 4 | 90.63% | 84.31% | CFLAR, PSEN1, CEACAM4, NAMPT, GZMK, NKTR, DUSP1, ITGAX, SLC25A37, RYBP, RXRA, EPOR, | | | | |  |  |  |  |
| 5 | 90.63% | 82.35% | CFLAR, MAPK9, PSEN1, CEACAM4, GZMK, NKTR, DUSP1, RARA, SLC25A37, RYBP, RXRA, EPOR, | | | | |  |  |  |  |
| 6 | 90.63% | 80.39% | CFLAR, PSEN1, CEACAM4, GZMK, NKTR, DUSP1, RARA, ITGAX, SLC25A37, RYBP, RXRA, EPOR, | | | | |  |  |  |  |
| 7 | 90.63% | 80.39% | CFLAR, MAPK9, PSEN1, CEACAM4, NAMPT, GZMK, NKTR, DUSP1, ITGAX, SLC25A37, RXRA, EPOR, | | | | |  |  |  |  |
| 8 | 90.63% | 80.39% | CFLAR, PSEN1, CEACAM4, NAMPT, GZMK, NKTR, DUSP1, RARA, ITGAX, SLC25A37, RYBP, EPOR, | | | | |  |  |  |  |
| 9 | 90.63% | 80.39% | CFLAR, PSEN1, CEACAM4, NAMPT, GZMK, NKTR, DUSP1, RARA, ITGAX, SLC25A37, RXRA, EPOR, | | | | |  |  |  |  |
| 10 | 90.63% | 78.43% | CFLAR, MAPK9, PSEN1, CEACAM4, GZMK, NKTR, DUSP1, RARA, ITGAX, SLC25A37, RXRA, EPOR, | | | | |  |  |  |  |
| 11 | 90.63% | 78.43% | CFLAR, MAPK9, PSEN1, CEACAM4, GZMK, NKTR, DUSP1, RARA, ITGAX, SLC25A37, RYBP, EPOR, | | | | |  |  |  |  |
| 12 | 90.63% | 78.43% | CFLAR, MAPK9, PSEN1, CEACAM4, NAMPT, GZMK, NKTR, ITGAX, SLC25A37, RYBP, RXRA, EPOR, | | | | |  |  |  |  |
| 13 | 90.63% | 78.43% | CFLAR, MAPK9, PSEN1, CEACAM4, NAMPT, GZMK, NKTR, DUSP1, RARA, ITGAX, SLC25A37, EPOR, | | | | |  |  |  |  |
