# Supplementary material for: The kSORT Assay to Detect Renal Transplant Patients at High Risk for Acute Rejection: Results of the Multicenter AART Study
Source: PLoS Med. 2014 Nov 11;11(11):e1001759. doi: 10.1371/journal.pmed.1001759 (PMC4227654; doi:10.1371/journal.pmed.1001759)
Supplement: Table S2 — The exact number of samples and patients used from each of the participating centers in the AART study. (DOCX) [file pmed.1001759.s007.docx]

**Supporting Table S2: The exact number of samples and patients used from each of the participating centers in the AART trial**

|  |  | **Total**  **P** | **Total**  **S** | **CPMC** (P/S) | **Emory** (P/S) | **UPMC** (P/S) | **UCLA** (P/S) | **Stanford** (P/S) | **Barcelona** (P/S) | **UCSF** (P/S) | **Mexico** (P/S) |
| --- | --- | --- | --- | --- | --- | --- | --- | --- | --- | --- | --- |
| **AART**  **143** | **AR** | 45 | 47 | 2/2 | 9/9 | 15/15 | 19/21 | / | / | / | / |
|  | **No-AR** | 90 | 96 | 21/25 | 9/11 | 49/51 | 9/9 | / | / | / | / |
|  |  | 135 | 143 |  |  |  |  |  |  |  |  |
| **AART124** | **AR** | 22 | 23 | 0/0 | 5/6 | 2/2 | 1/1 | 14/14 | / | / | / |
|  | **No-AR** | 85 | 101 | 7/8 | 14/16 | 11/13 | 12/13 | 41/51 | / | / | / |
|  |  | 107 | 124 |  |  |  |  |  |  |  |  |
| **AART100** | **AR** | 36 | 38 | / | / | 8/8 | / | / | 8/8 | 7/7 | 13/15 |
|  | **No-AR** | 60 | 62 | / | / | 13/13 | / | / | 8/8 | 31/33 | 8/8 |
|  |  | 96 | 100 |  |  |  |  |  |  |  |  |
| **AART191** | **AR** | 72 | 74 | / | 1/1 | 17/18 | 14/15 | 40/40 | / | / | / |
|  | **No-AR** | 199 | 216 | 33/37 | 30/34 | 57/61 | 28/33 | 51/51 | / | / | / |
|  |  | 271 | 290 |  |  |  |  |  |  |  |  |

**P =** patients; **S=** samples
